# Conceptualizing age‐appropriate social media to support children's digital futures

**DOI:** 10.1111/bjdp.70006

**Published:** 2025-07-11

**Authors:** Sonia Livingstone, Kim R. Sylwander

**Affiliations:** ^1^ Digital Futures for Children centre, London School of Economics and Political Science London WC2A 2AE UK

**Keywords:** cognitive development, children's rights, computers/technology, developmental theory, media, public/social policy, social/emotional development, social media

## Abstract

Is there really a ‘right age’ for social media? As governments rush to regulate children's digital lives, age‐based bans and ‘age‐appropriate’ design regulations are gaining international momentum. However, these are often based on theoretically dated ‘ages and stages’ models and blunt age thresholds. This article examines three seemingly divergent yet surprisingly convergent approaches. First, emerging regulatory frameworks are embedding ‘age‐appropriate’ design and bright‐line age limits. Second, social science research on children's digital experience, offers valuable documentation of developmental variability across ages but provides limited policy‐ready guidance and often lacks developmental theory. Third, a normative child rights framework grounded in the UN Convention on the Rights of the Child's principle of ‘evolving capacities’ urges a balance between protection and participation rights in ways that take into account children's variable capacities and increasing autonomy. Given the often fraught and contested nature of the debates over digital policy, we call on developmental psychologists to scrutinize proposed age thresholds, map developmental evidence to diverse contexts, and bring contemporary theory and robust evidence to inform policy. Without this input, decisions that matter to children's digital lives will be left to political expediency and corporate interests, overlooking or even undermining children's rights and developmental needs.

**Keypoints**

- Critiques the lack of a contemporary developmental psychological evidence base for age limits set in the regulation of children's social media use.
- Calls for a more nuanced approach that reflects children's diverse, context‐dependent developmental trajectories.
- Bridges developmental science with child rights through the principle of evolving capacities to approach age‐appropriate social media for children—supporting children's rights to protection, participation, and evolving autonomy in digital spaces.
- Exposes the gap between globalised digital policy and context‐specific child development.
- Calls for developmental psychologists to engage in digital policy debates and to inform the evidence base.

## IS THERE A RIGHT AGE FOR SOCIAL MEDIA?

At the end of 2024, the Australian ban on social media for under‐16s was passed by the Senate. Claims about child development popularly justified this—as young people over 16 are seen as ‘outside of the most vulnerable adolescent stage’ (Online Safety Amendment Bill, 2024). However, it was contested by childhood experts arguing for more nuanced approaches (Australian Child Rights Taskforce, 2024). Calls for similar bans are mounting internationally, as well as in the United Kingdom (BBC, 2025; McConvey, 2025). Again, generating popular support but contested by researchers on the grounds that evidence for social media harms is mixed, as is that for the effectiveness of bans, since children may find workarounds (EU Kids Online, 2024). When consulted, children often express concerns for their wellbeing but call for improved design and regulation for digital products and services to protect their safety and privacy. Far from being banned, they want to feel welcome in digital spaces and treated with respect, because they consider the ‘digital’ vital for their generation and future (Third & Moody, 2021).

Policies restricting children's access to social media (or smartphones, gaming or gambling) can seem a practical way for governments to safeguard children. However, it is becoming hard to determine the ‘right age’ (Livingstone & Sylwander, 2025), and restrictive regulation is being challenged both by big tech's massive lobbying power and by digital rights advocates concerned about freedom of speech and privacy. Policies to protect children are also contentious because, to know who is a child, platforms must subject all internet users to age assurance (the umbrella term for age verification, typically based on hard proof of legal identity, or ‘softer’ forms of age estimation, facial recognition or self‐declaration of age; Livingstone, Nair, et al., 2024). While age assurance technologies are improving, they rely on personal data, so they may infringe privacy, wrongly exclude certain groups or lead providers to withdraw services from children. Related proposals—for example, to assess users' competence or maturity (Laaber et al., 2024; Leahy & Dolan, 2010)—may compound such problems.

Faced with such challenges, many propose digital education as a viable alternative, or complement, to age‐based platform regulation, to ensure children can navigate the digital environment safely and enjoy its benefits. However, just as regulation relies, implicitly if not explicitly, on a developmental assessment of what is needed for children of different ages, the same can be said of digital education. Policymakers and educators must determine what children can be expected to understand or consent to at different ages, when they should be taught about what, and up to what age society should hold their parents and guardians responsible for children's protection (Ilomäki et al., 2023). Although children are undoubtedly gaining digital literacy, their knowledge is often insufficient to satisfy their own desires for information, education and communication or, crucially, cope with the complex interfaces, nudges and dark patterns of a highly commercial and increasingly automated data‐driven attention economy (5Rights, 2025).

The needs of a 5 and a 15‐year‐old are very different. In their journeys from birth to adulthood, children are frequently classified by age or maturity (e.g. infant, toddler, pre‐pubertal child, adolescent, etc.) for the purposes of parenting, welfare, health, education, justice and so on. This is rarely straightforward. In relation to the digital environment, such classification seems to many both vital and urgent. In this article, we explore the interface between developmental theory and evidence about children's development, social science research regarding their online risks and opportunities and the normative vision of the UN Convention on the Rights of the Child (UNCRC), which seeks to answer the question—what is the role of the state and other key actors in ensuring children's rights are respected, protected and fulfilled in diverse contexts, including online. We centre our analysis on three intersecting concepts—the social science research on child development, the child rights' emphasis on the evolving capacities of the child and the growing policy calls for age‐based or age‐appropriate platform design and regulation. Although the question of digital education is important, we here focus on building a rationale for social media regulation that takes into account children's age or development.

In what follows, we rely on the UNCRC's definition of a child as a person younger than 18 (or the age of majority in their country), noting that this is broadly consonant with the legal responsibilities of governments, educators and businesses to provide for ‘minors’. We recognize, however, that those under 18 may be described, and may describe themselves, using a range of terms—child, teenager, adolescent, young person or Gen Alpha and so on. Yet, the significance of such descriptions is rarely discussed. For sociologists, such labels may point to particular educational, parenting, peer culture or wider societal norms, whether protective or supportive of a constrained agency. For neuroscientists and many psychologists, what matters is brain development and its implications for cognitive competence, risk‐taking and other vulnerabilities (Blakemore, 2018). For historians and anthropologists, such labels are so substantially shaped by history (Cunningham, 2020) and culture (Lesko, 1996) as to permit few common assumptions about the meaning of age. Yet in relation to digital policy, multiple bright‐line rules draw particular and not always consistent or carefully evidenced lines between those under or over 18 years old, or maybe under or over 16 or 13 years old (Livingstone & Sylwander, 2025). Although the limitations of such rules and their implementation are increasingly recognized, the challenge is to identify what is appropriate for children of different ages or circumstances, as well as an evidence base to underpin such judgements.

## AGE‐BASED AND AGE‐APPROPRIATE POLICIES FOR SOCIAL MEDIA ARE BECOMING MORE COMMON

Certain forms of age‐based regulation are familiar, although not always effective. In many countries, it is illegal to provide under‐18s with pornography, gambling, drugs or knives, and what's illegal offline is said also to apply online. Social media companies typically set their minimum age at 13, influenced by the United States' 1998 Child Online Privacy Protection Act, which requires providers to seek verifiable consent from a parent or guardian before marketing to younger children or collecting their data. While this is not a ban on social media access by under‐13s, it has been taken as such by companies seeking to avoid the cost of contacting parents and is popularly referred to as ‘the digital age of consent’ (Montgomery & Koros, 2022). More recently, Europe's General Data Protection Regulation (GDPR) included a similar provision, again designed to protect young children's data, and again, social media companies took this as a minimum age of use, rather than taking steps to protect the privacy of younger children. In this instance, different member states set their own age limits between 13 and 16 (Lievens, 2020), though the rationale or evidence for such age limits is unclear. Moreover, such age limits are widely flouted by children and parents, while companies mainly turn a blind eye, leading child safety advocates to seek an alternative approach, asking whether platforms can be made ‘age‐appropriate’?

The UK's Data Protection Act, the Age‐Appropriate Design Code (AADC) (ICO, 2020), entered into force in 2021 and is enforced by the data regulator, the Information Commissioner's Office, to protect children's data and privacy. Known also as the Children's Code, it sets out 15 ‘standards of age‐appropriate design of relevant information society services which are likely to be accessed by children’ (ICO, 2020). Rather than bright‐line rules, the regulator explains to digital providers that the standards should be applied flexibly to protect all those under 18 (ICO, 2020, section 3). As the regulator explained to platforms, this ‘means that the age range of your audience and the different needs of children at different ages and stages of development should be at the heart of how you design your service and apply this code’. They further spell out five stages of child development: pre‐literate and early literacy (0–5); core primary school years (6–9); transition years (10–12); early teens (13–15); approaching adulthood (16–17) (ICO, 2020, Annex b).

Policy based on ‘ages and stages’ has an established history in digital policy in the UK, the United States, and elsewhere. The US Code defines age or developmentally appropriate as ‘activities or items that are generally accepted as suitable for children of the same chronological age or level of maturity or that are determined to be developmentally appropriate for a child, based on the development of cognitive, emotional, physical and behavioural capacities that are typical for an age or age group’ (42 USC § 675,11(a)). Without reprising the history of content regulation and advertising policy, both of which drew upon the then‐heyday of Piagetian developmental psychology, we will address the recent history. The UK Council for Child Internet Safety (2017) said of 10‐ to 13‐year‐olds, for instance, that these children have ‘the strong desire for immediate rewards triggers risk‐taking behaviour’, also pointing to possible increased risk‐taking due to the development of the pre‐frontal cortex in ages 14–18, as well as possible increased mental health issues and anxiety (p. 9). While the psychological evidence may be convincing, any link to digital activities or harms remains implicit. More closely tying child development to digital engagement and risk, Stoilova et al. (2019) conducted a systematic evidence review of children's online privacy to inform the regulator of children's emerging data literacy, influencing its implementation of the ICO's Children's Code (or AADC).

Much cited in such contexts is Kidron and Rudkin's (2023) Digital Childhood report. This aligns implicitly with developmental theorists like Piaget and Vygotsky, drawing on a range of empirical studies to describe the progression of children's capacities related to digital experiences. Integrating developmental concepts such as self‐regulation, critical awareness and online identity expression, it highlights children's age‐related vulnerabilities to persuasive design, peer pressure, misinformation and privacy risks. This helps to bridge or translate academic research for policymakers and practitioners for whom an ages and stages account of child development is reassuringly familiar and actionable. Indeed, for policymakers, age‐appropriate provision promises to facilitate the design of digital products and services in ways that fairly recognize children's developmental pathways, milestones or needs.

Age‐appropriate regulations are spreading across jurisdictions, as seen in the California Age‐Appropriate Design Code (CAADCA, 2022), the Dutch Code for Children's Rights (Code voor kinderrechten, 2022) and many more. Also influential, Europe's Digital Services Act (DSA) builds on standards such as Europe's CEN‐CENELEC Workshop Agreement 18,016 Age‐appropriate Digital Services framework (IEEE, 2023) in setting out the EU Code of Conduct for age‐appropriate design (European Commission, 2023). However, as the EU Kids Online network (2025) observed, the DSA guidelines address the needs of those under or over 18 but remain vague about the varying needs of children under 18.

Still, defining and implementing ‘age‐appropriate’ social media are challenging. It surely requires attention to the complex interaction between platform operation, design features, digital content and user social interaction across diverse circumstances. To provoke regulation, a series of whistleblowers have pointed out that Meta's products, for example, are highly unsuitable for children of different ages (Richard, 2023). Such provocations, combined with the success of the AADC, encourage child rights advocates such as the 5Rights Foundation (2025) to produce new guidance, for example, to make artificial intelligence systems ‘developmentally appropriate’. However, to resist regulation, platforms are hiring, arguably weaponizing, child development expertise in support of their defense that they already meet the needs of different age groups (Livingstone, Cantwell, et al., 2024).

Similarly, to implement the UK's 2023 Online Safety Act, the regulator reviewed research and evidence regarding ‘child development ages, stages and online behaviour’, concluding that ‘there is no perfect way to group children into developmental ages and stages. The most appropriate grouping is context‐dependent, and no age categories are absolute as children can develop at different rates’ (Ofcom, 2024, p. 3). Social media appears to raise questions for which academic consensus is lacking. For instance, at what age can or should platforms make a child's profile searchable, suggest new contacts to a child, permit them to see hateful content or escalate their indications of harm to a helpline?

## CAN DEVELOPMENTAL PSYCHOLOGY UNDERPIN AGE‐BASED AND AGE‐APPROPRIATE SOCIAL MEDIA REGULATION?

Developmental psychologists may be curious as to the origin of policy statements regarding the ages and stages of child development. They likely reflect some understanding of Piaget's theory of cognitive development, dominant half a century ago (Lourenço, 2016), though an explicit reference is rarely made. However, as is well known in developmental psychology, Piagetian theories have been widely criticized for being too reductive, mechanistic, individualistic, normative and more (although some argue that stage theories can be useful if interpreted as more heuristic or ‘fuzzy’; Lourenço, 2016; Winstanley, 2023). Contestation over the meaning of age and the implication of an underlying linear process of psychological development was most strongly made by the so‐called ‘new sociologists of childhood’ (James et al., 1998), although critical developmental psychology also joined the debate (Burman, 2007). Indeed, James et al.'s claim that ‘the immaturity of children is a biological fact of life but the ways in which this immaturity is understood and made meaningful is a fact of culture’ (p. 24) seems to erase the very possibility of a developmental psychology by skipping from biology to culture. Yet, within mainstream developmental psychology, there seems to be a silence about these critiques, along with a continued use of the vernacular language of ‘ages and stages’, albeit qualified by acknowledging individual variability and the importance of context (Davies, 2023).

A review of developmental theory post Piaget and Vygotsky paints a more dynamic picture. In tracing the trajectory of developmental psychology into developmental science over the past half century, Miller (2022) shows that past theories still influence current theorizing, even though current work favours less general and more domain‐specific theories (such as the development of a ‘theory of mind’ or reading or emotional regulation). However, current work is grounded less in mentalistic and more in biological perspectives, emphasizing how neurobiological, cognitive, emotional and sociocultural processes intersect across variable, interdependent and mutually influencing dynamic pathways, sometimes theorized as ‘developmental cascades’ (Masten & Cicchetti, 2010). Hence, ‘variability seems to be the rule, rather than the exception, and a child often performs differently, even on the same problem, from one time to another’ (Miller, 2022, p. 3).

Certainly, such variability is borne out by the evidence. Many countries regularly report on children's digital lives, often segmenting data by age to track trends in vulnerability and resilience across childhood. This shows, for instance, that younger children are using social media—in the United Kingdom, notwithstanding the platforms' minimum age of 13, half (51%) of children under 13 use social media −38% of 5‐ to 7‐year‐olds, rising to 63% of 8‐ to 11‐year‐olds and 92%–95% of 12‐ to 17‐year‐olds (Ofcom, 2024). Why does this matter? To inform the implementation of the UK's Online Safety Act, Bryce et al. (2023) conducted a Rapid Evidence Assessment of children's exposure to online sexual risks of harm applying a ‘risky pathways model [which] recognises that not all exposure to harmful contact or content has [negative] outcomes and highlights the importance of understanding the role of risk or vulnerability factors specific to the individual (e.g., depression, body image, family environment) in determining whether and how this occurs’ (p. 9). Although they found some evidence of age‐related vulnerabilities, the considerable variation in findings regarding exposure to risk and identification of its consequences made it hard to draw neat links between children's age and online risk of harm.

Similar conclusions can be drawn from a UNICEF‐commissioned rapid evidence review of the possible relation between children's online risks and opportunities and their well‐being (Stoilova et al., 2021). The review emphasized age as both a risk and a protective factor and reported little evidence of effective ways to support children's resilience. There was a distinct lack of evidence regarding younger children and of theory to link child development to children's digital experiences. Far more evidence documents how children's digital experiences are deeply shaped by offline circumstances, reflecting broader patterns of intersectionality (Alper et al., 2016). For example, middle‐class children often gain earlier and more comprehensive digital access than their less advantaged peers, which can expose them to certain risks as well as enhanced opportunities for digital literacy due to more supportive home environments (Stoilova et al., 2021). Comparing across high‐, middle‐ and low‐income countries, the range of risk and protective factors to be considered is magnified, especially where children face earlier expectations of responsibility, work, sexual behaviour and other cultural norms, along with a host of exclusions and disadvantages; all of these increasingly include a digital dimension and contribute to digitally mediated outcomes (Banaji et al., 2018; Ghai et al., 2022). Indeed, descriptive accounts applicable to the ‘average’ child in a usually implicit context (say, the urban middle‐class West) may seem plausible to policymakers on the face of it; for children in different contexts, they offer little explanatory or predictive value and thus provide only a weak rationale for policy intervention. Crucially, since differences are enormous across geographies, cultures, socioeconomics, accessibility, digital skills, digital cultures and much more, available findings may not only not be globally transferable, but overlooking the differences in situation and capacities between a 5‐ or 15‐year‐old in one culture compared with another may result in distinct injustices.

Meanwhile, in the United Kingdom, public policy increasingly references concerns that link adolescent social media use to declining mental health, calling for age‐based social media, with influential (social) psychologists like Jean Twenge (2019) and Jonathan Haidt (2024) leading the debate. Despite broad acceptance in popular discourse and public sentiment, empirical research on social media's effects remains inconclusive, producing mixed results and small effect sizes (Achterberg et al., 2022). Available longitudinal studies suggest developmental windows of sensitivity to social media during adolescence, with significant gender differences and bidirectional effects, and these will vary across geographies: Orben et al. (2022) found small effects of time spent using social media and subsequent life satisfaction a year later, notably among females aged 11–13 and males aged 14–15, coinciding with puberty for each gender. Such results suggest the need for gender‐differentiated age limits (much like the evidence regarding traffic accidents linked to age and gender) that are not easily translatable into policy.

Drawing on neurocognitive research, the idea that sensitivity to social media would be related to children's brain development is plausible, with its likes, follows, beauty standards, complicated contracts of consent and attention‐grabbing algorithms. Some research suggests social media use may affect brain development in the amygdala and pre‐frontal cortex during adolescence (important for emotional learning and behaviour, impulse control and emotional regulation). This suggests that the developing adolescent brain may be particularly sensitive to social media environments as they interact with the neural systems associated with social reward processing and emotional regulation (Crone & Konijn, 2018; Ladouceur et al., 2012). However, recent studies like Achterberg et al. (2022, p. 7) found minimal differences in brain anatomy between heavy and light social media users. The study highlights structural changes in socioemotional brain regions, such as the medial pre‐frontal cortex, during adolescence, suggesting a developmental window of heightened vulnerability. Small effect sizes linking social media use and poor mental health lead many to point to pre‐digital knowledge on how to promote healthy cognitive development among children and adolescents (Kern et al., 2014), which addresses environmental influences that also moderate media, particularly as offline vulnerabilities tend to be mirrored online (Orben et al., 2022; Valkenburg et al., 2022).

In short, the available research suggests that development plays a role in children's resilience, vulnerabilities and susceptibility to the risks and possible negative effects associated with social media use. However, it does not sufficiently support bright‐line rules or age limits, nor does it reliably inform recommendations on what is age‐appropriate. Moreover, a lack of theory about the possible relation between age and development means that the revival of ‘ages and stages’ in digital policy is neither supported nor contested. For policymakers, bright‐line age‐based rules may be easier to implement and enforce than contextual judgements about the maturity of particular children in particular circumstances. But the question remains as to whether ‘ages and stages’ policies for child online protection are acceptable, even valuable, or whether other theories and evidence would better guide policymaking.

## CHILDREN'S EVOLVING CAPACITIES: A CHILD RIGHTS APPROACH TO THEIR DIGITAL FUTURES

The psychology of child development is descriptive, theoretical and analytic, rather than normative. Translating developmental research into policy, through blunt age cut‐offs, too often overlooks children's diverse and evolving capacities. Therefore, a robust normative framework grounded in a child rights approach is needed. Hence, we turn to the principle of ‘evolving capacities’ articulated in Articles 5 and 14(2) of the UNCRC to ground our child rights approach to development and age. The advantages of a child rights approach include that it is holistic, as children's rights are indivisible and interdependent, and should not be ranked, cherry‐picked or traded off against each other. Second, the UNCRC has been ratified by states worldwide, and being international soft law is, in various ways, being incorporated into national (hard) law and policy. Third, a child rights approach is child‐centred (though not child‐centric), respecting and recognizing children's place in the world and avoiding adultism (Wall, 2022). Fourth, while not naively universalist, it prioritizes common understandings of what matters for children, while acknowledging contextual variation in implementation. Fifth and last, it is practical, bringing a host of tried‐and‐tested tools and processes of child rights and human rights assessment and due diligence that are recognized by states, businesses and civil society, including in relation to the digital world where ideas of child‐rights‐by‐design are gaining ground (Livingstone & Pothong, 2023).

Eekelaar (1994) articulated evolving capacities as ‘dynamic self‐determinism’, aiming to balance children's rights to protection and autonomy. He argued that paternalistic interpretations of the ‘best interests of the child’ principle risk reducing children to passive objects of protection. Conversely, he argues, simply recognizing children's ‘right to be heard’ falls short of fully acknowledging their agency. In a 2016 general comment, the UN Committee on the Rights of the Child (2016, para 18) defined evolving capacities as ‘an enabling principle that addresses the process of maturation and learning through which children progressively acquire competencies, understanding and increasing levels of agency to take responsibility and exercise their rights’. Lansdown (2005) similarly emphasizes that as children develop new competencies, a child rights approach requires that they should be supported progressively to obtain autonomy so as to exercise their rights independently.

The principle of evolving capacities, therefore, supports children's full spectrum of rights, being grounded in the presumption of autonomy in human rights law. Introduced during the UNCRC's drafting before its adoption in 1989, the principle was controversial due to tensions between protection and autonomy (Varadan, 2019). As an emancipatory or enabling principle, it insists that adults (parents and caregivers, educators, professionals, states and digital service providers) should enable children's exercise of positive rights and freedoms as early as possible—including through supported (or scaffolded) decision‐making, while maintaining protections throughout childhood—ideally up to 18 (Lansdown, 2005). This means that all rights apply to all children irrespective of the ‘acquisition of their autonomy’.

The principle of evolving capacities acknowledges that children develop in diverse, non‐linear ways, with the significance of ‘age’ varying across cohorts, individuals and rights. The principle respects both the child's present state (of ‘being’) and their future development (of ‘becoming’), ensuring neither is overshadowed and resisting privileging protection over freedom, a common tendency in regulating the digital environment. Originally, UNCRC drafters viewed children's limited capacity as an obstacle to claiming rights beyond protection and education. Yet, full autonomy, especially for younger children, was also implausible. Tobin (2015) suggests resolving this tension by anchoring rights not in capacity but in the human interests universally vested in children and adults alike. In taking such ideas forward, Daly and Walsh (2025) have critiqued the UN Committee on the Rights of the Child for its focus on early childhood and adolescence, largely overlooking middle childhood. This matters since middle childhood is characterized by crucial emerging capacities and increased responsibilities. As Peleg and Varadan (2025) argue, this omission is especially significant in today's digital context as children navigate online environments with greater independence, often without parental supervision. Put positively, by recognizing children's diverse developmental paths across cultures and contexts, the principle of evolving capacities can underpin the legitimacy of concepts such as age‐appropriate design.

It remains the case, as Todres and Kilkelly (2025a, p. 2) observe, that there has been little analysis of how the UNCRC ‘might differ in application to a 5‐year‐old compared with a 15‐year‐old’ or ‘what steps duty bearers—notably governments—should take to ensure the implementation of children's rights law in developmentally appropriate ways for children of all ages’. They suggest this lack of analysis is due to the lack of communication between child rights and child development experts. Their edited volume combines evidence from neurobiology with that of emotional, linguistic and social development over the years from birth to adulthood, contextualised in relation to society's provision for children of different ages. The result is a portrait of children's changing competences, interests, dependencies and vulnerabilities in the face of empirical opportunities or disadvantages. At best, they provide a compilation of relevant theories that, in combination, help account for the differences between the 5‐ and 15‐year‐old, avoiding the commitment to a strict ages and stages approach but tolerant of descriptors that group children by fuzzy categories such as early or middle childhood. Such context‐specific descriptions may be the best that research can currently manage, particularly in the fast‐changing digital environment.

As the UNCRC's principles were developed in a pre‐digital age, the Committee's General comment No. 25 (2021) outlines how children's rights apply in the digital environment. It recognizes that the principle of evolving capacities has particular significance in the digital environment as children can ‘engage more independently from supervision by parents and caregivers’ (para. 19), and that the risks and opportunities children face in the digital environment will vary across development and age (ibid.). These considerations should guide age‐appropriate design of measures to protect as well as facilitate children's access to the digital environment. Implementing these requirements isn't easy on an individual basis and is even harder for children as a collective, as is often required in relation to the provision and regulation of digital services. The principle of evolving capacities challenges the current fashion for bans and bright‐line age rules—for a ban represents the opposite of enabling, and undermines the important expectation of facilitating positive rights from as early as possible while extending protections as longer as they are needed. Moreover, unlike those favouring bans, advocates of a child rights approach would avoid the implied binary that offline is good and online is bad, for this overlooks the nuanced reality of children's integrated digital lives.

Bright‐line age limits are set for a number of activities, services and legal responsibilities—such as age of sexual maturity, driving, marriage, alcohol consumption and criminal liability—in ways that are considered acceptable to children's rights and often in favour of the protection of children's rights (Livingstone & Sylwander, 2025). Yet, these limits often lack a robust child rights foundation. Rap (2025) observes that age limits intended to navigate tensions between children's protection and autonomy often fail to clearly substantiate their appropriateness or effectiveness (see also Eekelaar, 1994). This is particularly relevant to children's digital engagement, which encompasses diverse and complex interactions, such as social interaction with friends, data collection by companies, vulnerability to the attention economy, bullying, exposure to explicit content and more. Todres and Kilkelly (2025b) also highlight inconsistencies in the UNCRC's guidance on developmental ages and maturity (noting the right to be heard and age of criminal responsibility), noting a lack of consensus and uniform application about ages and stages among both child development research and children's rights. They advocate incorporating developmental science more explicitly in applying children's rights law, challenging the adequacy of the traditional age‐of‐eighteen cut‐off in light of neuroscientific evidence, arguing for a more developmentally responsive and empirically based child rights approach to age limits.

On the one hand, child rights advocates need to encompass digital dilemmas. For instance, while there is extensive jurisprudence on determining the age of unaccompanied migrant children to treat them according to their evolving capacity and in ways that respect their rights (to privacy, protection, voice, remedy, etc.; Quan & Skelton, 2025), the equivalent challenge of assuring the age of a child online also raises questions of privacy, protection, access to information and participation (Livingstone, Nair, et al., 2024). On the other hand, state and business practices of managing children's access to digital resources of all kinds need to build the capacity to recognize and address the many child rights issues that arise (UNICEF, 2024). The legitimacy of both, we have argued, rests significantly on the underpinning account of children's development from infancy to adulthood. Although this is rarely articulated, nor is developmental psychology much cited, questions about how children's digital lives are best managed remain hotly debated (Livingstone & Sylwander, 2025).

## CONCLUSIONS

In this article, we first reviewed recent policy efforts to make social media ‘age‐appropriate’ for children. Concerned that it could be left to businesses to define what is appropriate for children of digital ages or maturity, we focused on two lines of inquiry to identify a possible basis for these policy efforts—first, developments in developmental theory, accompanied with empirical research on children's digital experiences linked to age and development, and second, a child rights approach to respecting children's evolving capacities. Intriguingly, and importantly, it would seem that these lines of inquiry are converging. Post‐Piagetian developmental science and the child rights emphasis on evolving capacity share an understanding of development as processual, variable, relational and multiply influenced according to context. For each, the task is to understand the biographical and contextually specific experiences of children as agents who, individually and collectively, influence and are influenced by the actions of others in their environment.

But this leaves both science and law at odds with the seeming policy and practice imperative to draw the kinds of bright‐line age‐based rules which tacitly assume an internal and invariant developmental logic that unfolds inexorably in a linear fashion independent of context. Our intent in this paper has been, on the one hand, intellectual—to understand how research can conceptualize and research children's digital experiences across the span of childhood from infancy to adulthood. On the other hand, it has been normative and future‐oriented—we are keen to discover whether developmental science can help resolve the present situation of discursive confusion and uncertainty, in which academics, clinicians, experts and pundits argue among themselves about whether social media engagement is beneficial or harmful for children, including which children and at what age. Worryingly, the more fraught these arguments, the more the authority of researchers, parents and educators is undermined, while the easier digital platforms find it to undermine political and regulatory efforts to rein them in.

The mutual articulation of three distinct concepts is at issue, comprising the practical realm of regulation, which focuses on making social media platforms *age‐appropriate*; the normative realm of law and rights, especially child rights, where the key concept is that of children's *evolving capacities* (Article 5, UNCRC); and research on psychology and wellbeing, where what matters is healthy *child development*. Reconciling these poses a challenging agenda. Noting that developmental theory is largely implicit, missing or dated when referenced by empirical studies on the effects of social media, and noting that social media policy to protect children often lacks an empirical evidence base, we call on developmental psychologists to engage in these debates, bringing both their theory and evidence as well as undertaking new targeted investigations. These are especially vital insofar as research and policy are constantly playing catch‐up as technological developments career ahead. Such investigations could map current evidence to the proposed or actual age limits in digital policy, asking, does it support the proposed age, or would the evidence point to a different age or age range? It is important to specify the applicability of findings to different cohorts or contexts or subgroups, to identify the error (and possible injustices) that might result from an age limit—on whom, and in what circumstances, might the costs of the policy fall significantly? Such research should be conducted across diverse contexts, recognizing how policies may be widely applied, and with a focus on the experiences of those who are marginalized, vulnerable or disadvantaged. Also valuable would be to propose age‐appropriate forms of mitigation or remedy for when the age limits designed to prevent risk of harm introduce risks of their own. More radically, should developmental psychologists argue against top‐down age‐based interventions and propose alternative ways of safeguarding children and realizing their rights? To be effective in policy circles, such developmental psychology could partner with economists to calculate the pros and cons ‐ and costs ‐ of age‐based decisions. On the other hand, in line with Article 12 of the UNCRC on children's right to be heard in matters that affect them—there is merit in a child‐centred participatory research agenda that ensures developmental perspectives are grounded in children's lived experiences in the digital environment, not least to make sure that researchers are aware of what children are doing and experiencing.

Although we have focused primarily though not exclusively on influential developments in the United Kingdom and Europe, social media platforms are owned by major multinational corporates operating in many markets. Policies to ensure they protect rather than harm children are also influenced by transnational forms of regulation, especially as regard online safety, privacy and data protection laws. Consequently, child development expertise should recognize cross‐cultural and contextual differences as well as similarities. As most children and young people live in the global South, yet most research on children's digital experiences is conducted in the global North, there is considerable potential for injustice when this research informs policies that spread globally (e.g. the AADC) or guides child safeguarding by companies with global markets (notably, Meta, TikTok, Google and many others) (Draper et al., 2023; Ghai et al., 2022; Sirin et al., 2024). Determining what is age‐appropriate must be considered globally to avoid imposing Western norms of child development onto digital policymaking, which affects the global South. While developmental psychology has long called for more cross‐cultural research, the rapid advance of global digital companies can result in policies judged age‐appropriate in one country being extended too widely. Responding effectively to these calls is now imperative. If developmental psychologists refrain from entering such debates, others will not, and populist concerns and business interests will continue to set the agenda.

## AUTHOR CONTRIBUTIONS

**Sonia Livingstone:** Conceptualization; methodology; writing – original draft; writing – review and editing. **Kim R. Sylwander:** Conceptualization; methodology; writing – original draft; writing – review and editing.

## CONFLICT OF INTEREST STATEMENT

The authors declare no conflicts of interest.

## Data Availability

Data sharing not applicable to this article as no datasets were generated or analysed during the current study.
